# Quality and Readability of ChatGPT and Gemini Responses to Patient Questions About Systemic Isotretinoin: Comparative Evaluation Study

**DOI:** 10.2196/99780

**Published:** 2026-10-30

**Authors:** Elif Bal Avcı, Buğra Burç Dağtaş, Dilara İlhan Erdil, Ozan Erdem, Andaç Salman, Selçuk Korkmaz, Sezgi Sarıkaya Solak

**Affiliations:** 1 Department of Dermatology, Faculty of Medicine, Trakya University, Trakya University Balkan Campus, Iskender, Edirne, Türkiye, 90 5054511134; 2 Department of Dermatology, Istanbul Training and Research Hospital, Istanbul, Türkiye; 3 Department of Dermatology, Etlik City Hospital, Ankara, Türkiye; 4 Department of Dermatology, Medeniyet University Faculty of Medicine, Göztepe Prof. Dr. Suleyman Yalcin City Hospital, Istanbul, Türkiye; 5 Department of Dermatology, School of Medicine, Acıbadem Mehmet Ali Aydınlar University, Istanbul, Türkiye; 6 Department of Biostatistics and Medical Informatics, Trakya University Faculty of Medicine, Edirne, Türkiye

**Keywords:** artificial intelligence, AI, large language models, patient education, readability, systemic isotretinoin

## Abstract

**Background:**

The use of AI in health care is rapidly expanding due to its accessibility, with growing reliance among both physicians and patients.

**Objective:**

This study evaluated ChatGPT (OpenAI) and Gemini (Google) responses to patient questions on systemic isotretinoin.

**Methods:**

A total of 40 commonly anticipated patient questions were developed based on online sources and patient education materials and categorized into 5 groups. Responses were generated using the January 2025 free versions of ChatGPT (GPT-4o) and Gemini 2.0 Flash. A total of 5 experienced dermatologists evaluated the responses in a blinded manner using a 5-point Likert scale assessing accuracy, clarity, completeness, usefulness, and overall quality score (Global Quality Score [GQS]). Readability was measured using Flesch-Kincaid Grade Level (FKGL), Flesch Reading Ease, and Simple Measure of Gobbledygook (SMOG) index scores. Mixed-effects modeling was performed to adjust for reviewer and question variability, and intraclass correlation coefficients (ICCs) were calculated to assess interrater reliability.

**Results:**

ChatGPT achieved significantly higher ratings in accuracy, completeness, usefulness, and GQS, while clarity showed a nonsignificant trend. When questions were analyzed by subtype, no statistically significant differences were observed between models across evaluation parameters. Mixed-effects modeling confirmed higher odds of superior ratings for ChatGPT across domains (odds ratios [ORs] 1.82‐5.88; all *P*<.001). However, interrater reliability was lower for ChatGPT (global ICC 0.401) compared with Gemini (global ICC 0.677). Readability analyses demonstrated that ChatGPT required a higher reading level (FKGL 12.6 vs 11.2; SMOG 11.3 vs 10.0).

**Conclusions:**

ChatGPT outperformed Gemini in terms of data accuracy and quality, while Gemini offered superior readability and consistency. Both models exceeded recommended readability thresholds, highlighting a gap between informational quality and linguistic accessibility in AI-generated health communication.

## Introduction

Systemic isotretinoin is among the most effective treatments for moderate-to-severe acne [1]. However, its use involves significant adverse effects, strict monitoring, and complex counseling. As a result, patients frequently seek detailed information on efficacy, safety, laboratory monitoring, teratogenic risks, and long-term outcomes [2]. As health information becomes increasingly digitalized, patients are increasingly using online sources to supplement information provided by physicians [3]. AI fulfills diverse roles in dermatology, such as facilitating diagnosis, monitoring disease progression, guiding treatment selection, providing patient information, and supporting medical education [4,5]. Numerous studies across various medical specialties have used AI language models to evaluate responses to patient questions. However, standardized guidelines for performance assessment are lacking, and the methodological details of these studies often remain unclear [6]. Several studies have assessed the performance of large language models (LLMs) in responding to patient questions about acne and systemic isotretinoin, with mixed results for accuracy and quality [7-11]. Due to isotretinoin’s teratogenicity, monitoring needs, and medicolegal considerations, it is essential that the information provided is both accurate and easily understandable.

The study aimed to comparatively evaluate ChatGPT and Gemini in their responses to patient questions regarding systemic isotretinoin, assessing performance across multiple quality domains, interrater reliability, and readability metrics.

## Methods

### Study Design

This cross-sectional comparative study assessed the quality of responses generated by 2 LLMs to patient-oriented questions concerning systemic isotretinoin. The evaluation criteria included accuracy, clarity, completeness, usefulness, Global Quality Score (GQS), and readability.

### Question Development

Forty patient-oriented questions were developed based on information from patient information brochures and online patient forum queries related to isotretinoin. To prevent confusion between topical and systemic formulations, the standardized term “oral isotretinoin” was included in each question. The questions were categorized into 5 domains: general information, efficacy and treatment process, side effects, follow-up and precautions, and cosmetic concerns. Six experienced dermatologists (EBA, BBD, OE, DİE, AS, and SSS) reviewed and validated all questions to ensure content relevance and representativeness prior to inclusion. The complete list of the 40 isotretinoin-related questions evaluated in this study is provided in Multimedia Appendix 1.

### Evaluation of AI Models and Query Procedures

Free access versions of Gemini 2.0 Flash (Google Inc) and GPT-4o (OpenAI) were evaluated through their standard web-based interfaces to reflect real-world patient use. All queries were conducted in January 2025. The models were not accessed via APIs; therefore, API-level parameters, including temperature, were not accessible or configurable by the investigators. Each question was entered verbatim, without additional prompts or role instructions. Queries were submitted without logging into a user account, using incognito browsing mode, and each question was asked in a separate browser session to minimize the influence of prior conversational context. Each question was submitted only once to each model, and the first generated response was used for evaluation, with no regeneration or repeated querying, to simulate a typical real-world patient interaction. Responses were recorded verbatim without modification.

### Blinded Expert Assessment (Accuracy, Clarity, Completeness, Usefulness, and the GQS)

The questions were entered into the 2 chatbots by 1 dermatologist (EBA) to generate the responses, and the resulting outputs were independently evaluated by 5 experienced dermatologists (≥5 years of experience; BBD, OE, DİE, AS, and SSS) in a blinded fashion. Interrater agreement was assessed using the intraclass correlation coefficient (ICC). Responses were scored on a 5-point Likert scale (1=very poor, 5=excellent) across 5 domains: accuracy, clarity, completeness, usefulness, and the GQS. A standardized scoring rubric was provided to evaluators prior to assessment. Evaluations were performed by 5 experienced dermatologists, informed by contemporary acne treatment guidelines and clinical practice standards. Readability was assessed using Readability Formulas [12]. The following indices were calculated for each response: Flesch Reading Ease (FRE), Flesch-Kincaid Grade Level (FKGL), and Simple Measure of Gobbledygook (SMOG) index. Source citation was not included as a comparative parameter because the reference provision differed structurally between models: Gemini frequently generated citations, while ChatGPT did not consistently provide explicit references.

### Statistical Analysis

Data were analyzed using R (version 4.3.2; R Foundation for Statistical Computing). Descriptive statistics are presented as mean, median, and range (minimum-maximum), as appropriate. Ratings assigned to ChatGPT and Gemini responses to the same questions by the same reviewer panel were compared using the Wilcoxon signed-rank test for paired samples. These comparisons were performed for the pooled dataset and separately within each of 5 predefined clinical domains (general information, efficacy and treatment process, side effects, follow-up and precautions, and cosmetic concerns), resulting in 30 pairwise comparisons (5 metrics across 6 analyses). Within each analysis, Bonferroni correction was applied across the 5 metric-level comparisons (adjusted *P*=min[raw *P* × 5, 1.0]) to control the family-wise error rate. A Bonferroni-adjusted *P*<.05 was considered statistically significant. To account for the hierarchical structure of the ratings and repeated assessments by evaluators and across questions, model performance for accuracy, clarity, completeness, usefulness, and GQS was additionally evaluated using ordinal mixed-effects regression models, with evaluator and question included as random intercepts. Effect estimates are reported as odds ratios (ORs) with 95% CIs. Interrater reliability was assessed using 2-way mixed-effects ICCs for absolute agreement. All tests were 2-sided, with a significance level set at *P*<.05.

### Ethical Considerations

This study did not involve human participants, animal subjects, or patient data. The analyzed data consisted solely of responses generated by publicly available AI chatbots to questions prepared by the authors. Therefore, ethics committee approval was not required.

## Results

### Expert Assessment of AI Responses

ChatGPT demonstrated consistent numerical outperformance across all assessed domains (Table 1).

In the domain-specific analysis, no statistically significant differences between ChatGPT and Gemini were detected across the 5 predefined domains (general information, efficacy and treatment process, side effects, follow-up and precautions, and cosmetic concerns) for any of the assessed metrics after Bonferroni adjustment (all adjusted *P*>.05). Although ChatGPT showed numerically higher mean scores across all metrics in the cosmetic concerns domain, including accuracy (4.60 vs 3.80) and completeness (4.60 vs 3.60), these differences were not statistically significant after adjustment. The complete results of the domain-specific comparisons are presented in Table 2.

**Table 1.** Performance comparison of ChatGPT and Gemini across key quality metrics.

Metric	ChatGPT, mean (SD; range)	ChatGPT, median (IQR)	Gemini, mean (SD; range)	Gemini, median (IQR)	*P* value	Adjusted *P* value
Accuracy	4.37 (0.71; 3-5)	5 (4-5)	4.20 (0.65; 3-5)	4 (4-5)	.004	.02
Clarity	4.42 (0.72; 3-5)	5 (4-5)	4.27 (0.62; 3-5)	4 (4-5)	.01	.07
Completeness	4.39 (0.75; 3-5)	5 (4-5)	3.77 (0.75; 1-5)	4 (3-4)	<.001	<.001
Usefulness	4.42 (0.70; 3-5)	5 (4-5)	4.04 (0.69; 2-5)	4 (4-4)	<.001	<.001
GQS^a^	4.42 (0.71; 3-5)	5 (4-5)	4.05 (0.70; 2-5)	4 (4-5)	<.001	<.001

a GQS: Global Quality Score.

**Table 2.** Domain-specific comparison of performance metrics between ChatGPT and Gemini across 5 predefined domains.

Domain and metric	ChatGPT, mean (SD; range)	ChatGPT, median (IQR)	Gemini, mean (SD; range)	Gemini, median (IQR)	*P* value	Adjusted *P* value
General information						
Accuracy	4.00 (0.00; 4-4)	4 (4-4)	4.00 (0.00; 4-4)	4 (4-4)	—^a^	—
Clarity	4.00 (0.00; 4-4)	4 (4-4)	4.33 (0.58; 4-5)	4 (4-4.5)	>.99	>.99
Completeness	3.67 (0.58; 3-4)	4 (3.5-4)	3.33 (0.58; 3-4)	3 (3-3.5)	>.99	>.99
Usefulness	4.00 (0.00; 4-4)	4 (4-4)	4.00 (0.00; 4-4)	4 (4-4)	—	—
GQS^b^	4.00 (0.00; 4-4)	4 (4-4)	4.00 (0.00; 4-4)	4 (4-4)	—	—
Efficacy and treatment process						
Accuracy	3.89 (0.33; 3-4)	4 (4-4)	3.56 (0.53; 3-4)	4 (3-4)	.15	.75
Clarity	4.00 (0.71; 3-5)	4 (4-4)	4.00 (0.50; 3-5)	4 (4-4)	>.99	>.99
Completeness	3.78 (0.44; 3-4)	4 (4-4)	3.44 (1.01; 1-4)	4 (3-4)	.37	>.99
Usefulness	3.89 (0.33; 3-4)	4 (4-4)	3.67 (0.71; 2-4)	4 (4-4)	.35	>.99
GQS	3.78 (0.44; 3-4)	4 (4-4)	3.56 (0.73; 2-4)	4 (3-4)	.35	>.99
Side effects						
Accuracy	3.82 (0.40; 3-4)	4 (4-4)	3.91 (0.30; 3-4)	4 (4-4)	.77	>.99
Clarity	3.91 (0.30; 3-4)	4 (4-4)	4.09 (0.70; 3-5)	4 (4-4.5)	.48	>.99
Completeness	3.91 (0.54; 3-5)	4 (4-4)	3.55 (0.52; 3-4)	4 (3-4)	.20	>.99
Usefulness	3.91 (0.30; 3-4)	4 (4-4)	3.82 (0.40; 3-4)	4 (4-4)	.77	>.99
GQS	4.00 (0.00; 4-4)	4 (4-4)	3.91 (0.54; 3-5)	4 (3-4)	.77	>.99
Follow-up and precautions						
Accuracy	4.00 (0.43; 3-5)	4 (4-4)	4.00 (0.00; 4-4)	4 (4-4)	>.99	>.99
Clarity	4.50 (0.52; 4-5)	4.5 (4-5)	4.50 (0.52; 4-5)	4.5 (4-5)	>.99	>.99
Completeness	4.25 (0.45; 4-5)	4 (4-4.25)	3.92 (0.29; 3-4)	4 (4-4)	.07	.36
Usefulness	4.25 (0.45; 4-5)	4 (4-4.25)	4.08 (0.29; 4-5)	4 (4-4)	.42	>.99
GQS	4.50 (0.52; 4-5)	4.5 (4-5)	4.33 (0.49; 4-5)	4 (4-5)	.53	>.99
Cosmetic concerns						
Accuracy	4.60 (0.55; 4-5)	5 (4-5)	3.80 (0.45; 3-4)	4 (4-4)	.07	.36
Clarity	4.40 (0.55; 4-5)	4 (4-5)	3.80 (0.45; 3-4)	4 (4-4)	.15	.75
Completeness	4.60 (0.55; 4-5)	5 (4-5)	3.60 (0.89; 2-4)	4 (4-4)	.09	.45
Usefulness	4.60 (0.55; 4-5)	5 (4-5)	3.80 (0.45; 3-4)	4 (4-4)	.07	.36
GQS	4.60 (0.55; 4-5)	5 (4-5)	3.80 (0.45; 3-4)	4 (4-4)	.07	.36

a *P* value was not calculated because there was no variation in the corresponding scores.

b GQS: Global Quality Score.

Ordinal mixed-effects models revealed that ChatGPT was significantly more likely than Gemini to receive higher ratings across all metrics (all *P*<.001). ChatGPT outperformed Gemini in accuracy (OR 1.94, 95% CI 1.28‐2.94), clarity (OR 1.82, 95% CI 1.19‐2.76), and GQS (OR 3.55, 95% CI 2.32‐5.43). The most substantial advantages were observed in completeness (OR 5.88, 95% CI 3.84‐9.02) and usefulness (OR 3.74, 95% CI 2.44‐5.74). Representative examples of responses generated by ChatGPT and Gemini are provided in Multimedia Appendix 2.

### Interrater Reliability

Global ICC estimates indicated “moderate” consistency for Gemini (ICC 0.677) and “poor” consistency for ChatGPT (ICC 0.401; Table 3). Although both models exceeded chance agreement (*P*<.05), Gemini demonstrated significantly more stable evaluator consensus.

**Table 3.** Comparison of interrater reliability (intraclass correlation coefficient [ICC]) between ChatGPT and Gemini across key evaluation domains.

Metric	ChatGPT, ICC (95% CI)	Gemini, ICC (95% CI)	Interpretation (ChatGPT)	Interpretation (Gemini)
Accuracy	0.435 (0.101‐0.671)	0.554 (0.291‐0.740)	Poor	Moderate
Clarity	0.389 (0.028‐0.644)	0.629 (0.410‐0.784)	Poor	Moderate
Completeness	0.379 (0.012‐0.638)	0.527 (0.248‐0.725)	Poor	Moderate
Usefulness	0.393 (0.035‐0.647)	0.593 (0.353‐0.763)	Poor	Moderate
GQS^a^	0.462 (0.145‐0.687)	0.636 (0.421‐0.788)	Poor	Moderate
Global ICC	0.401 (0.048‐0.651)	0.677 (0.486‐0.812)	Poor	Moderate

a GQS: Global Quality Score

### Readability

Gemini demonstrated superior accessibility across multiple readability metrics (Table 4). Significant reductions in grade-level requirements (mean 11.2 vs 12.6; adjusted *P*=.002) and SMOG index scores (adjusted *P*=.001) were observed for Gemini compared with ChatGPT. Although Gemini also achieved higher raw FRE scores, this difference did not reach statistical significance after Bonferroni adjustment (*P*=.09).

**Table 4.** Comparison of readability indices between ChatGPT and Gemini.

Index	ChatGPT, mean (SD; range)	ChatGPT, median (IQR)	Gemini, mean (SD; range)	Gemini, median (IQR)	*P* value	Adjusted *P* value
FRE^a^	37.9 (10.26; 17-72)	38.00 (31.00-42.25)	42.03 (12.62; 20-65)	42.5 (32.00-51.75)	.03	.09
FKGL^b^	12.62 (1.95; 7.7-16.59)	12.44 (11.48-13.51)	11.16 (2.13; 7.15-15.14)	10.805 (32.00-51.75)	<.001	.002
SMOG^c^	11.31 (1.65; 7.78-15.37)	11.31 (10.40-12.10)	10.03 (1.63; 7.24-13.62)	9.865 (8.79-11.12)	<.001	.001

a FRE: Flesch Reading Ease.

b FKGL: Flesch-Kincaid Grade Level.

c SMOG: Simple Measure of Gobbledygook.

## Discussion

In this study, ChatGPT demonstrated significantly higher performance across evaluation metrics, including accuracy, completeness, clarity, and overall quality, while Gemini showed greater interrater reliability and superior readability. Accordingly, the choice of LLM may depend on whether the priority is high-quality medical content or improved patient comprehension.

Recent evaluations indicate that LLMs generate more contextually appropriate medical responses than traditional internet-based resources [13]. ChatGPT has demonstrated potential as a public dermatology resource, particularly for common dermatological diseases, with 88% of its responses rated as appropriate [14]. However, the clinical utility of LLMs remains constrained by their propensity to generate fictitious citations [15,16]. Their inability to account for individual patient factors and age-related nuances may also lead to misleading guidance, potentially resulting in adverse outcomes based on unverified data [11].

The literature further demonstrates that LLM accuracy and comprehensiveness ratings vary considerably across dermatologic diseases and topics [11]. For example, ChatGPT’s accuracy has ranged from 36% to 90% across various topics [6,14,17,18]. ChatGPT showed higher accuracy in dermatological topics with well-established evidence, such as melanoma and nonmelanoma skin cancers, rosacea, and alopecia. In contrast, lower accuracy was observed in areas requiring individualized approaches or where scientific consensus remains limited, including the effects of diet on skin diseases, nonpharmacological treatments for eczema and psoriasis, antiaging interventions, skincare routines, and wound healing [14]. In a study examining isotretinoin-related queries, ChatGPT’s accuracy varied by domain: daily life achieved 72% reliability, while the treatment process yielded 64% with outdated or incorrect data [7]. In contrast, our findings showed no significant differences across query categories.

Our findings contradict previous research indicating that Gemini provided more accurate answers than ChatGPT on isotretinoin questions [8], as well as studies asserting Gemini’s superiority in comprehensiveness in melanoma-related queries [18]. Recent evidence regarding Mohs surgery indicates that GPT-4 outperforms Gemini (Google Bard) in clinical relevance and accuracy, providing answers that closely align with official American College of Mohs Surgery recommendations [19], suggesting that differences in topic may significantly influence the performance of LLMs. Furthermore, such discrepancies may result from the absence of standardized evaluation criteria and differences among LLM models and versions.

Consistent with prior research on isotretinoin, which reported a low Fleiss κ (κ=0.045) [7], our study also demonstrated similarly weak interrater agreement. Consistent with our findings, Gemini demonstrated higher interrater agreement than ChatGPT in a study of common dermatological conditions [20]. This low interrater agreement may be partially attributed to heterogeneity in physicians’ clinical communication styles and expectations in patient education. To address inconsistent evaluations, it has been proposed that LLM outputs be assessed using standardized, guideline-based frameworks [21]. Nevertheless, the complex dynamics of physician-patient interactions and the subjective nature of clinical judgment continue to influence evaluation outcomes.

Previous studies suggest that AI-generated health information often exceeds recommended readability levels, potentially limiting accessibility for individuals with low health literacy [21-23]. Recent studies have shown that successive iterations of LLMs exhibit progressive improvement in generating more readable and accessible content [24,25]. In our study, both ChatGPT and Gemini produced patient information at readability levels exceeding recommended thresholds for public education materials. Consistent with our results, some studies report higher readability for Gemini [18], whereas others find no significant readability differences between Gemini and ChatGPT [8]. High readability demands, incomplete incorporation of updated approaches, and limited actionable guidance preclude the use of LLM outputs as standalone patient education tools [7,8]. For high-risk medications such as isotretinoin, patients should be explicitly informed that LLM-generated outputs constitute preliminary information and do not substitute for professional medical guidance. Given the substantial patient concerns about isotretinoin [26-28], clear and accessible educational materials may mitigate misconceptions, reduce treatment resistance, and improve adherence.

Additionally, ChatGPT can be directed to create patient brochures at predefined reading levels, thereby improving accessibility for diverse patient populations [18,29]. Effective prompt engineering enables LLMs to deliver clearer, more targeted responses [8,30-32]. Incorporating an audience-adaptive response model in which users identify as health care professionals or laypersons prior to output generation could facilitate flexible adjustment of terminology and grade-level complexity.

Gemini consistently advised consultation with a health care professional, whereas this was not observed in our study with ChatGPT, despite prior reports of such disclaimers [33,34]. This suggests that Gemini may offer a comparatively safer profile for patient safety.

The strengths of this study include methodological transparency, a comprehensive multidimensional evaluation approach, a comparative design, and the provision of actionable recommendations. This study has several limitations. The selected questions may not accurately represent real-world patient inquiries. The study was conducted exclusively in English, and language-dependent variability in AI model training data and source retrieval may limit generalizability across different languages. Moreover, despite blinded assessment, clinicians familiar with AI language models may have inferred the model’s identity, potentially introducing observer bias. Another limitation is that our analysis included only ChatGPT and Gemini. Therefore, the findings may not be generalizable to other commercially available or open-source LLMs. Given the rapid evolution of LLMs, the findings of this study reflect the performance of GPT-4o and Gemini 2.0 Flash at the time of evaluation. As newer model iterations are released with enhanced reasoning and language capabilities, ongoing comparative studies are required to determine whether these advancements yield measurable improvements in clinical quality, reliability, and readability. Furthermore, because LLMs are frequently updated, their responses and performance may change over time. Future studies should include a broader range of models and evaluate changes across model versions longitudinally.

In conclusion, there is a clear distinction between ChatGPT’s technical accuracy and Gemini’s linguistic accessibility. For these models to function as reliable clinical tools, transparent source attribution and patient-centered, intelligible outputs are imperative. Adopting standardized, objective evaluation frameworks is also crucial for enabling robust comparisons of outputs and tracking model performance over time, thereby mitigating interrater variability. However, the complexity and heterogeneity of patient characteristics make it unlikely that clinicians will be fully substituted for by AI, particularly when it comes to contextualizing clinical evidence for individual patients.

## Supplementary material

10.2196/99780 Multimedia Appendix 1 List of questions.

10.2196/99780 Multimedia Appendix 2 Example questions and answers.

## Abbreviations

FKGL: Flesch-Kincaid Grade Level
FRE: Flesch Reading Ease
GQS: Global Quality Score
ICC: intraclass correlation coefficient
LLM: large language model
OR: odds ratio
