# Supplementary material for: Quality and Readability of ChatGPT and Gemini Responses to Patient Questions About Systemic Isotretinoin: Comparative Evaluation Study
Source: JMIR Dermatol. 2026 Oct 30;9:e99780. doi: 10.2196/99780 (PMC13721848; doi:10.2196/99780)
Supplement: Supplementary file 1 [file derma-v9-e99780-s001.docx]

**GENERAL INFORMATION**

1)     What is oral isotretinoin?

2)     How does oral isotretinoin work to treat acne?

3)     How is oral isotretinoin different from other acne treatments?

**EFFICACY AND TREATMENT PROCESS**

4)     How long does it take to see results from oral isotretinoin?

5)     Will my acne come back after I stop taking oral isotretinoin?

6)     How should I take oral isotretinoin?

7)     How long will I need to take oral isotretinoin?

8)     Why do some people get worse acne at first when they start oral isotretinoin?

9)     What should I do if I miss a dose while taking oral isotretinoin?

10) Can I use other acne treatments while taking oral isotretinoin?

11) Can I take oral isotretinoin with other medications?

12) Can oral isotretinoin be taken for a second course?

**SIDE EFFECTS**

13) What are the common side effects of oral isotretinoin?

14) Is there a difference in side effect if I take oral isotretinoin a lower dose or a higher dose?

15) Can oral isotretinoin cause any permanent side effects?

16) How can I manage the dryness caused by oral isotretinoin?

17) Does oral isotretinoin affect my fertility?

18) Can oral isotretinoin cause depression or suicidal thoughts?

19) Can oral isotretinoin cause joint or muscle pain?

20) How does oral isotretinoin affect my liver?

21) Can oral isotretinoin affect my cholesterol?

22) Can oral isotretinoin cause hair thinning or loss?

23) Can oral isotretinoin cause problems with my vision?

**FOLLOW-UP AND PRECAUTIONS**

24) Can oral isotretinoin cause inflammatory bowel disease?

25) Is oral isotretinoin safe for teenagers, or are there age restrictions?

26) How often do I need to see my doctor while taking oral isotretinoin?

27) Which lab tests should I have during oral isotretinoin treatment?

28) Should I avoid the sun or use sunscreen while taking oral isotretinoin?

29) Is oral isotretinoin safe during pregnancy or breastfeeding?

30) How long should I wait to get pregnant after stopping oral isotretinoin?

31) What is the iPledge program, why do I neet it for oral isotretinoin treatment?

32) Can I drink alcohol while taking oral isotretinoin?

33) Should I avoid any foods or supplements while taking oral isotretinoin?

34) Can I donate blood while taking oral isotretinoin?

35) Can I exercise while taking oral isotretinoin?

**COSMETIC CONCERNS**

36) Can I wear makeup while taking oral isotretinoin?

37) Can I get cosmetic procedures while on oral isotretinoin?

38) When can I restart skincare treatments after stopping oral isotretinoin?

39) Can oral isotretinoin help with hyperpigmentation or redness after acne?

40) Can oral isotretinoin help with acne scars?
