# Supplementary material for: Quality and Readability of ChatGPT and Gemini Responses to Patient Questions About Systemic Isotretinoin: Comparative Evaluation Study
Source: JMIR Dermatol. 2026 Oct 30;9:e99780. doi: 10.2196/99780 (PMC13721848; doi:10.2196/99780)
Supplement: Supplementary file 2 [file derma-v9-e99780-s002.docx]

| **Domain** | **Representative patient question** | **GPT-4o response** | **Gemini 2.0 Flash response** |
| --- | --- | --- | --- |
| **GENERAL INFORMATION** | What is oral isotretinoin? | Oral isotretinoin is a medication primarily used to treat severe acne, particularly cystic acne, that hasn't responded to other treatments such as antibiotics or topical medications. It is a form of vitamin A and works by reducing the size of sebaceous (oil) glands in the skin, leading to a decrease in oil production. This helps prevent clogged pores, which can contribute to acne.  Isotretinoin also has anti-inflammatory effects and reduces the growth of the bacteria that contribute to acne. It is typically prescribed for cases of severe acne that are resistant to other treatments or when acne is causing significant scarring.  Due to its potency, isotretinoin requires close monitoring by a healthcare provider. It has several potential side effects, including dry skin, lips, and eyes, and can lead to more serious side effects such as liver damage or birth defects if taken during pregnancy. As a result, people taking oral isotretinoin are often required to participate in a risk management program (such as the iPLEDGE program in the United States) to prevent pregnancy and monitor for side effects.  The treatment is usually taken for several months, and many patients experience significant improvement in their acne, sometimes with long-lasting results. However, it is important to follow a healthcare provider's instructions carefully to minimize risks and ensure the best outcome. | This is for informational purposes only. For medical advice or diagnosis, consult a professional.  Oral isotretinoin is a medication used to treat severe acne. It is a retinoid, which means it is a derivative of vitamin A. Isotretinoin works by reducing the size of oil glands in the skin, which helps to prevent the formation of pimples. It also helps to unclog pores and reduce inflammation.  Isotretinoin is a very effective medication for treating severe acne, but it can have serious side effects. It is important to talk to your doctor about the risks and benefits of isotretinoin before taking it.  Here are some of the potential side effects of isotretinoin:  * Dry skin, lips, and eyes  * Nosebleeds  * Muscle aches  * Headaches  * Increased sensitivity to the sun  * Depression  * Birth defects (if taken during pregnancy)  If you are considering taking isotretinoin, it is important to talk to your doctor about the risks and benefits of this medication. |
| **EFFICACY AND TREATMENT PROCESS** | How long does it take to see results from oral isotretinoin? | The time it takes to see results from oral isotretinoin (commonly known by brand names like Accutane) can vary depending on the individual, but most people begin to notice improvements within 4 to 6 weeks of starting treatment. However, full results typically take longer—around 3 to 6 months. Here's a more detailed timeline of what to expect:  First 1-2 months: Many people experience an initial worsening of their acne. This is often referred to as a "purge," where the skin gets worse before it gets better as the isotretinoin works to bring acne to the surface.  3-4 months: By this time, most patients start to see significant improvement. Pustules and cysts may begin to shrink, and the overall number of breakouts usually starts to decrease.  5-6 months: For many individuals, this is when the full effects of isotretinoin are most noticeable. Acne tends to clear up substantially, with fewer new breakouts, and existing scars may begin to heal.  Post-treatment: After finishing the course (which typically lasts 4 to 6 months), most people experience long-term clearance, but some may require a second course depending on their individual response. Important Notes: The duration of treatment and the dose vary from person to person. Your doctor will determine the appropriate length and dosage based on your acne's severity and how you respond. Some patients might experience dryness, irritation, or other side effects, which usually improve with time. Results can sometimes continue to improve for up to a year after completing the treatment. Always follow your dermatologist's instructions carefully, as isotretinoin has potential side effects and requires monitoring throughout the treatment. | This is for informational purposes only. For medical advice or diagnosis, consult a professional.  It can take several weeks or months to see results from oral isotretinoin. Some people may see improvement in their acne as early as 4 weeks after starting treatment, but it usually takes longer to see the full effects. A typical course of isotretinoin lasts about 15 to 20 weeks (4 to 5 months), and you may continue to see improvement throughout this time.  Here are some things to keep in mind:  • Everyone responds differently to isotretinoin. Some people may see results sooner than others.  • It may take several months to see the full effects of isotretinoin. Even if you see some improvement early on, it may take several months for your acne to clear up completely.  • Your doctor will monitor your progress and adjust your dose as needed.  If you are taking isotretinoin and are not seeing any results after several months, talk to your doctor. They may want to adjust your dose or try a different medication. |
| **SIDE EFFECTS** | What are the common side effects of oral isotretinoin? | Oral isotretinoin (commonly known by brand names like Accutane, Roaccutane, or others) is a potent medication used primarily to treat severe acne, particularly cystic acne that does not respond to other treatments. While it can be highly effective, it also carries a risk of various side effects. Common side effects of oral isotretinoin include:  1. Skin and mucous membrane dryness  • Dry skin: This is the most common side effect. Isotretinoin reduces the size and output of sebaceous (oil) glands, leading to drier skin.  • Chapped lips: Almost everyone who takes isotretinoin will experience dry or cracked lips.  • Dry eyes: Can cause discomfort, especially for contact lens wearers.  • Dry nose: This can lead to nosebleeds due to dryness of the nasal passages.  2. Changes in mood or mental health  • Depression and anxiety: There have been reports linking isotretinoin use to mood changes, including depression, anxiety, and in rare cases, suicidal thoughts. Monitoring for any mental health changes is important during treatment.  3. Joint and muscle pain  • Arthralgia (joint pain): Some people report muscle or joint pain during treatment. This can be temporary but can be severe for some individuals.  4. Gastrointestinal issues  • Nausea: Some people experience mild gastrointestinal discomfort, including nausea.  • Diarrhea: This is less common but can occur, and there have been occasional reports of more severe gastrointestinal problems.  5. Changes in liver function and lipid levels  • Elevated liver enzymes: Isotretinoin can affect liver function, so liver function tests (LFTs) are regularly monitored during treatment.  • Increased cholesterol and triglycerides: The medication may cause increases in blood lipid levels, particularly triglycerides. This is usually monitored with blood tests.  6. Hair thinning  • Some people experience mild hair thinning or shedding, though this is generally temporary and resolves after stopping the medication.  7. Photosensitivity (Increased sensitivity to sunlight)  • People taking isotretinoin may become more sensitive to sunlight, leading to sunburn more easily. It's important to use sunscreen and protective clothing.  8. Teratogenicity (Birth defects)  • Severe birth defects: Isotretinoin is highly teratogenic, meaning it can cause severe birth defects if taken during pregnancy. Women who are pregnant or planning to become pregnant must not take isotretinoin. It is recommended that women use two forms of contraception and undergo regular pregnancy tests while on the medication.  9. Eye problems  • Some individuals may experience irritation, dryness, or other eye-related issues like blurry vision, particularly with contact lenses.  10. Other potential effects  • Headaches: Some people experience headaches, and in rare cases, intracranial hypertension (increased pressure in the brain), which can cause severe headaches, blurred vision, and other symptoms.  • Rash: A mild rash can develop in some people.  • Changes in bone density: Long-term use may affect bone density, though this is typically seen with prolonged high-dose therapy.  Monitoring and Precautions:  Due to the potential side effects, regular blood tests are required during isotretinoin therapy to monitor liver function, lipid levels, and other markers. Additionally, mental health should be closely monitored, and any signs of depression, mood changes, or unusual thoughts should be reported to a healthcare provider immediately.  Most side effects tend to improve or resolve after stopping the medication, but some individuals may experience long-term effects. Always consult with a healthcare provider before starting or discontinuing isotretinoin. | This is for informational purposes only. For medical advice or diagnosis, consult a professional.  Isotretinoin is a medication commonly used to treat severe acne. It can be very effective, but it also comes with some potential side effects. Here are some of the most common side effects of oral isotretinoin:  • Dryness: This is one of the most common side effects and can affect the skin, lips, eyes, nose, and mouth.  • Skin sensitivity: Isotretinoin can make your skin more sensitive to sunlight, so it's important to use sunscreen with an SPF of 30 or higher every day.  • Muscle and joint pain: Some people experience muscle aches or joint pain while taking isotretinoin.  • Nosebleeds: Nosebleeds are also a common side effect.  • Headache: Headaches can occur in some people taking isotretinoin.  • Mood changes: In rare cases, isotretinoin can cause mood changes, such as depression or anxiety.  This is not an exhaustive list of all possible side effects. It's important to talk to your doctor about any concerns you have before starting isotretinoin. They can discuss the potential risks and benefits of the medication with you.  Please note that this information is for general knowledge and informational purposes only and does not constitute medical advice. Consult a qualified healthcare professional for diagnosis and treatment of any medical conditions. |
| **FOLLOW-UP AND PRECAUTIONS** | Can oral isotretinoin cause inflammatory bowel disease? | Oral isotretinoin, commonly prescribed for severe acne, has been associated with various gastrointestinal side effects, but its direct link to inflammatory bowel disease (IBD), such as Crohn's disease or ulcerative colitis, remains a topic of ongoing research and debate.  Potential Connection:  Some studies and case reports have suggested a possible association between isotretinoin use and the development of IBD. The theory is that isotretinoin may alter the gut microbiome, affect mucosal immunity, or disrupt the epithelial barrier, leading to an inflammatory response that could trigger or exacerbate IBD in susceptible individuals.  However, the evidence remains inconclusive, and most studies do not show a definitive cause-and-effect relationship. Some findings include:  • Case Reports: A number of isolated case reports have documented individuals developing IBD shortly after starting isotretinoin treatment, but these are anecdotal and do not prove causality.  • Large-Scale Studies: Larger studies, such as cohort studies, have generally not found a strong, consistent link between isotretinoin and IBD. For example, a study published in the Journal of the American Academy of Dermatology found no increased risk of IBD among isotretinoin users compared to the general population.  • Other Research: Some research has suggested a small, potential risk, particularly among individuals with a family history of gastrointestinal issues, but these findings have been inconsistent.  Risk Factors:  If there is any risk, it may be higher in individuals with a personal or family history of gastrointestinal diseases, including IBD, or those who are genetically predisposed to such conditions. In these cases, the risk-benefit ratio of using isotretinoin may need to be carefully considered by the prescribing doctor.  Conclusion:  While oral isotretinoin has been associated with a few cases of IBD, the overall risk appears to be low and the evidence inconclusive. If you have concerns or a family history of inflammatory bowel disease, it's important to discuss these with your healthcare provider before starting isotretinoin. If IBD symptoms (such as persistent diarrhea, abdominal pain, or weight loss) occur while on isotretinoin, medical attention should be sought promptly. | This is for informational purposes only. For medical advice or diagnosis, consult a professional.  There is no definitive answer to whether oral isotretinoin can cause inflammatory bowel disease (IBD).  Some studies have suggested a possible link between isotretinoin use and the development of IBD, particularly Crohn's disease. However, other studies have not found a significant association.  More research is needed to determine the exact relationship between isotretinoin and IBD.  If you are taking isotretinoin and experience symptoms of IBD, such as abdominal pain, diarrhea, or rectal bleeding, talk to your doctor. |
| **COSMETIC CONCERNS** | Can I wear makeup while taking oral isotretinoin? | Yes, you can wear makeup while taking oral isotretinoin (commonly known as Accutane), but there are some important things to consider for your skin's health during treatment:  1. Use Non-Comedogenic Products  Isotretinoin can make your skin more sensitive and prone to dryness, so it's crucial to choose makeup that is non-comedogenic (meaning it won't clog pores). Look for products labeled as such, as they are formulated to be less likely to cause breakouts.  2. Moisturize Well  Isotretinoin can cause dryness, so it's important to moisturize regularly. A good hydrating moisturizer will help create a smoother base for makeup application. Consider a hydrating primer that also offers some protection for sensitive skin.  3. Gentle Removal  Since your skin may become more sensitive, it's essential to remove makeup gently at the end of the day. Opt for a gentle makeup remover or micellar water instead of harsh cleansers that could further irritate your skin.  4. Avoid Heavy or Thick Layers  While it’s okay to wear makeup, try to avoid heavy layers, especially foundation or concealer that could exacerbate dryness or clog pores. Mineral makeup or lighter formulations can be better choices.  5. Be Mindful of Sun Protection  Isotretinoin makes the skin more sensitive to sunlight, so always apply sunscreen before makeup, ideally a broad-spectrum SPF 30 or higher. Some foundations have SPF, but they shouldn't be your only line of defense.  In summary, makeup is fine while on isotretinoin, as long as you choose gentle, non-comedogenic, and hydrating products, and focus on skincare routines that support the health of your skin. If you have specific concerns, it's always a good idea to check in with your dermatologist for personalized advice. | This is for informational purposes only. For medical advice or diagnosis, consult a professional.  Yes, you can wear makeup while taking oral isotretinoin (Accutane). In fact, makeup can help cover up redness and dryness that may occur as side effects of the medication. However, it's important to choose oil-free, non-comedogenic makeup that won't clog your pores. You should also avoid harsh cleansers and exfoliants that can further dry out your skin.  Here are some tips for wearing makeup while on isotretinoin:  • Use a gentle, fragrance-free cleanser to remove your makeup.  • Choose oil-free, non-comedogenic makeup that won't clog your pores.  • Avoid harsh cleansers and exfoliants that can further dry out your skin.  • Moisturize your skin well before applying makeup.  • Apply sunscreen daily, even on cloudy days.  If you have any concerns about wearing makeup while on isotretinoin, talk to your doctor. |
